# Effects of DAPT and Atoh1 Overexpression on Hair Cell Production and Hair Bundle Orientation in Cultured Organ of Corti from Neonatal Rats

**DOI:** 10.1371/journal.pone.0023729

**Published:** 2011-10-20

**Authors:** Li-Dong Zhao, Wei-Wei Guo, Chang Lin, Li-Xian Li, Jian-He Sun, Nan Wu, Li-Li Ren, Xin-Xin Li, Hui-Zhan Liu, Wie-Yen Young, Wei-Qiang Gao, Shi-Ming Yang

**Affiliations:** 1 Department of Otolaryngology Head and Neck Surgery, Institute of Otolaryngology, Chinese PLA General Hospital, Beijing, China; 2 Department of Otolaryngology Head and Neck Surgery, First Affiliated Hospital of Fujian Medical University, Fuzhou, China; 3 Renji-MedX Clinical Stem Cell Research Center, Renji Hospital, Shanghai Jiao Tong University School of Medicine, Shanghai, China; 4 Med-X Research Institute, Shanghai Jiao Tong University, Shanghai, China; 5 Shanghai Cancer Institute, Renji Hospital, Shanghai Jiao Tong University School of Medicine, Shanghai, China; Instituto de Medicina Molecular, Portugal

## Abstract

**Background:**

In mammals, hair cells do not undergo spontaneous regeneration when they are damaged and result in permanent hearing loss. Previous studies in cultured Organ of Corti dissected from neonatal animals have shown that both DAPT (r-secretase inhibitor in the Notch signal pathway) treatment and Atoh1 overexpression can induce supernumerary hair cells. The effects of simultaneous DAPT treatment and Atoh1 over expression in the cells of cultured Organ of Corti from neonatal rats are still obscure.

**Principal Findings:**

In this study, we set out to investigate the interaction of DAPT treatment and Atoh1 overexpression as well as culture time and the location of basilar fragment isolated form neonatal rat inner ear. Our results showed that DAPT treatment induced more hair cells in the apical turn, while Atoh1 overexpression induced more extra hair cells in the middle turn of the cultured Organ of Corti. When used together, their effects are additive but not synergistic. In addition, the induction of supernumerary hair cells by both DAPT and Atoh1 overexpression is dependent on the treatment time and the location of the cochlear tissue. Moreover, DAPT treatment causes dramatic changes in the orientation of the stereociliary bundles of hair cells, whereas Atoh1 overexpression didn't induce drastic change of the polarity of stereociliary bundles.

**Conclusions/Significance:**

Taken together, these results suggest that DAPT treatment are much more potent in inducing supernumerary hair cells than Atoh1 overexpression and that the new hair cells mainly come from the trans-differentiation of supporting cells around hair cells. The orientation change of stereociliary bundle of hair cells may be attributed to the insertion of the newly formed hair cells. The immature hair bundles on the newly formed hair cells may also contribute to the overall chaos of the stereociliary bundle of the sensory epithelia.

## Introduction

The mammalian Organ of Corti is composed of sensory cells (i.e., inner and outer hair cells, IHCs and OHCs) and supporting cells, and it is very precisely assembled in a mosaic distribution pattern, to which the Notch signaling pathway makes a great contribution [1]. The sensory hair cells in the inner ear can be easily damaged by many factors such as ototoxic drugs, noise exposure and ischemia, resulting in sensory neural hearing loss. Hearing loss in birds and amphibians is transient and can be fully restored because the hair cells in their inner ears can be regenerated through trans-differentiation or mitosis from supporting cells [2], [3], [4], [5], [6], [7], [8], [9], [10]. However, sensory neural hearing loss is permanent and currently incurable in mammals because the hair cells in mammalian cochleae cannot be regenerated spontaneously [11], [12], [13].

Hair cell regeneration may be one of the best ways to restore hearing. Many breakthrough discoveries have advanced this field in recent years. For example, as a member of the basic helix-loop-helix family and a pro-hair cell gene, Math1(the mouse homolog of drosophila Atoh1) has been shown to be necessary for the development and differentiation of hair cells [14]. During the most important period of hair cell differentiation, Math1 expression begins as early as E12.5 and continues until P0. Math1 knockout mice fail to produce cochlear or vestibular hair cells [15]. Over-expression of the Math1 gene in the inner ears of postnatal rats can induce robust production of extra hair cells, which trans-differentiate from great epithelial ridge cells and supporting cells in the utricle [16]. When adult animals are deafened by aminoglycoside treatment, adenoviral Math1 delivery to non-sensory cells induces the production of new hair cells [17]. On the other hand, the Notch signaling pathway plays an important role in determining hair cell and supporting cell fate through lateral inhibition during inner ear development. The Math1 gene lies downstream of the Notch signaling pathway. Activation of the Notch signaling pathway leads to the expression of Hes1 and Hes5, which in turn inhibit Math1 gene expression. Blockade of the Notch pathway by delivering the r-secretase inhibitors MDL28170 and DAPT to cultured neonatal Organ of Corti results in down regulation of the Hes1 and Hes5 genes. This down regulation releases the Math1 promoter and promotes Math1 expression in supporting cells, thereby producing supernumerary hair cells [18], [19].

Although the administration of r-secretase inhibitors and the overexpression of Math1 can increase the number of hair cells, their interaction when both are used simultaneously has remained uninvestigated. In the present study, we cultured Organ of Corti dissected from newborn rats and treated them with DAPT (an r-secretase inhibitor) and adinovirus-Atoh1-EGFP overexpression both simultaneously and separately. Our results demonstrated that DAPT treatment and overexpression of the Atoh1 gene induce the formation of extra hair cells in an additive but not synergistic manner. Furthermore, the increase in the number of OHCs during the treatment depended on their location and culture time, and we observed that DAPT treatment changed the orientation of stereociliary bundles dramatically. However, Atoh1 over-expression did not markedly change the polarization of the stereociliary bundles. These results suggest that Atoh1 and DAPT act differently in the development of the stereocilia bundles on hair cells, even though they both can induce the production of extra hair cells.

## Results

**Figure 1:**
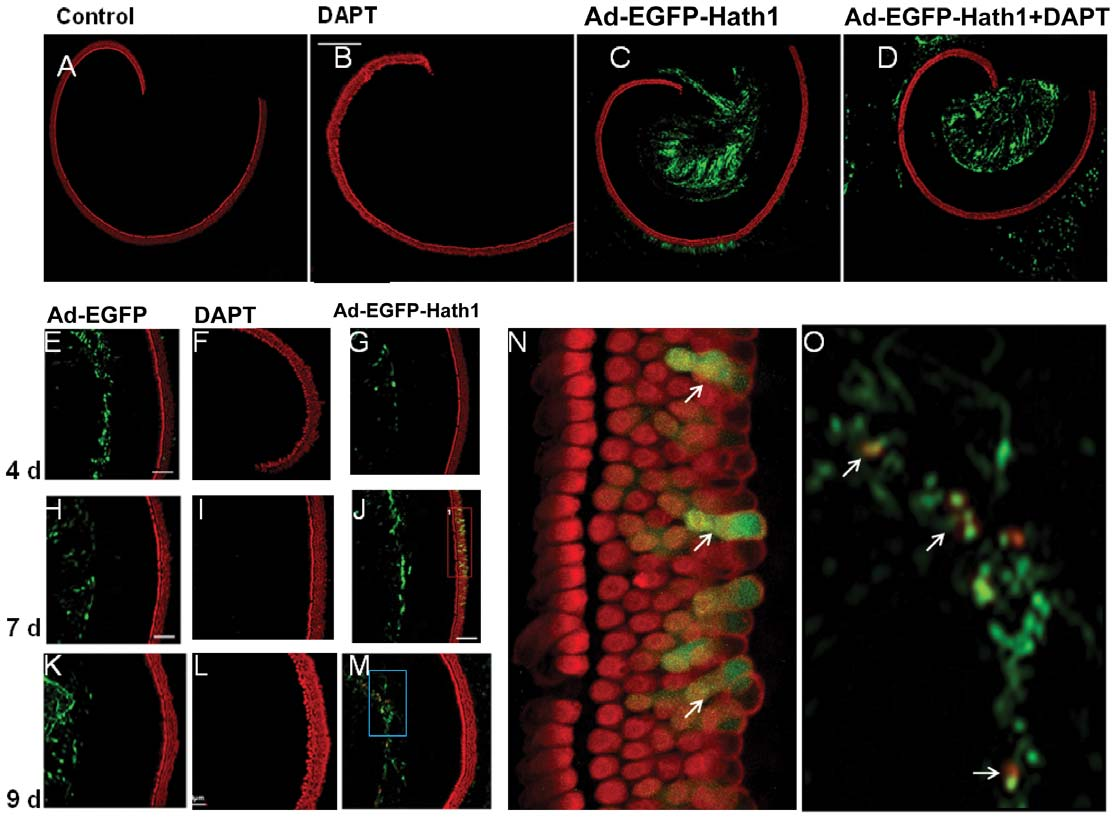
The overexpression of EGFP and Atoh1 in the Organ of Corti. A–D: Cultured Organ of Corti whole mounted on the bottom of the Petri dish in vitro. A: Control sample without treatment. B: With DAPT treatment. C: With ad-EGFP-Atoh1 overexpression. D: With ad-EGFP-Atoh1 overexpression and DAPT treatment. E–M: parts of the cultured basilar membrane for 4–9 days. E, H, K: Cultured basilar membranes transfected with adenovirus that carrying EGFP gene for 4, 7, and 9 days, indicating the expression of EGFP in the region of great epithelia ridge (GER). F, I, L: Cultured basilar membranes treated with DAPT for 4, 7, and 9 days. G, J, N: Cultured basilar membranes transfected with adenovirus that carrying EGFP and Atoh1 gene for 4, 7, and 9 days. N, Enlarged picture in the red Frame in panel J. white arrows indicating the extra hair cells express Myosin VIIa and EGFP in the Organ of Corti. O: Enlarged picture in the blue Frame in panel M (indicating the cells express Myosin VIIa and EGFP in GER region (white arrows). Green channel: EGFP expression. Red channel: Myosin VIIa expression.

We paid special attention in isolating and stretching the basilar membrane as Abdouh et al [20] have reported that extra rows of outer hair cells might appear spontaneously in vitro. Consequently, the Organ of Corti from the P0 rats were successfully isolated and stretched well on the bottom of the culture dish.No considerable hair cells loss happened during the isolation (fig. S1 A–L). In the control group, the Organ of Corti consisted of one row of IHCs and three rows of OHCs. All of the OHC and IHC are neatly arranged. No apparent hair cell loss was observed, which indicates that the basilar membrane and the Organ of Corti were kept intact in the isolated basilar fragments (60/65 cultured fragments) (Fig. S1). In the Atoh1 and DAPT+Atoh1 groups, adv-Atoh1-EGFP transfection was successful in all of the cultured basilar membrane fragments (30 observations/30 transfected fragments). EGFP expression was observed in the great epithelial ridge cells and in the spiral ganglion cell region. Non-specific Myosin VIIa or EGFP antibody binding was not observed (Fig. S1 G–L). Some cells in the Organ of Corti expressed EGFP (Fig. 1C, D).

### 1. DAPT treatment can significantly increase the number of IHCs and OHCs in cultured Organ of Corti from P0 rats

**Figure 2:**
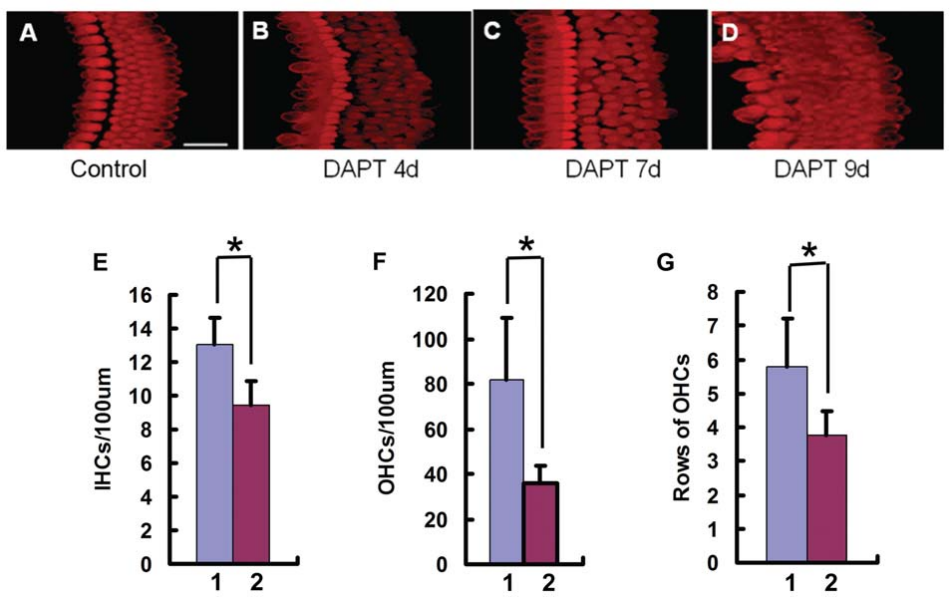
DAPT treatment significantly increases the number of IHCs and OHCs in the cultured P0 rat Organ of Corti. A: Control culture without treatment; B–D: The cultured Organ of Corti treated with DATP for 4, 7 and 9 days. E, F and G: Cell counts of IHCs and OHCs, as well as the rows of OHCs in the cultures. In the abscissa, 1 means with DAPT treatment, 2 means c.

**Figure 3:**
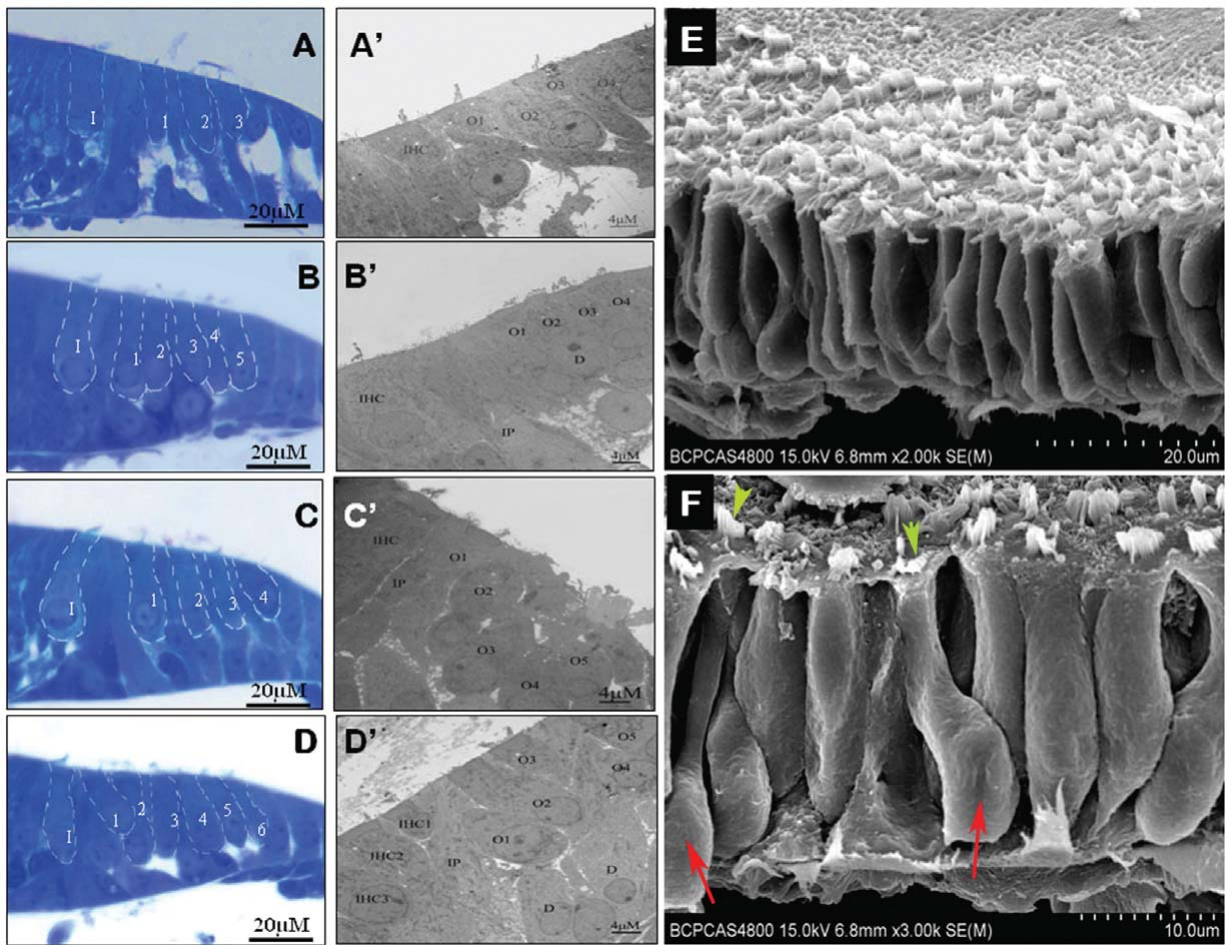
The semi-thin (A, B, C, D) and ultrastructural micrographs (A′, B′, C′, D′) of the cochlear epithelia. A, A′: Control culture; B, B′: Cultured Organ of Corti infected with adeno-Atoh1-EGFP; C, C′: Cultured Organ of Corti treated with DATP; D, D′: Cultured Organ of Corti with Atoh1 over expression plus DAPT treatment. E and F: Scanning electron microscopic image of the DAPT treated Organ of Corti. F: the enlarged picture of another optical field showing the lateral view of the cultured sensory epithelia. There were two cells (red arrows) whose bodies are very similar to that of Deiters' cells, but there were also hair bundles (green arrow heads) on their top face, which might indicate that the extra hair cells came from the trans-differentiation of immature Deiters' cells.

To investigate the effects of r-secretase inhibition on hair cell differentiation, we added DAPT (final concentration 5 µM) to cultured Organ of Corti isolated from newborn rats (P0) from the start of the in vitro culture and collected specimens for hair cell counting at 4, 7 and 9 days. Under the confocal microscope, the OHCs in the control group were kept in 3 regular rows (Fig. 2A). In the DAPT treated group, myosin VIIa-positive OHCs increased from 3 rows to 4 or 5 rows, even 7 or 8 rows (Fig. 2A–D). In the specimens that had been cultured for 7 or 9 days, the gap between the IHCs and the OHCs was narrower. The OHCs were crowded together and out of order (Figs. 1B; 2 A–D; 3 C, C′, E and F). Statistical analysis showed that the numbers of IHCs, OHCs, and rows of OHCs increased significantly in 10 DAPT-treated rats compared to those without DATP treatment (10 rats, p<0.0001) (Fig. 2E–G). To investigate the origin of the newly appearing hair cells induced by DAPT treatment, we prepared scanning electron microscope specimens presenting a lateral view of the hair cells. The results showed that all the hair cells were crowded together. Athough no Deiters' cells could be observed (Fig. 3E, F), there were some cells' bodies that were similar to that of Deiter's cells and with stereocialliary bundles on the top, which suggests that the Deiters' cells had been trans-differentiated into hair cells induced by the DAPT treatment.

### 2. Atoh1 overexpression could induce extra OHCs in the middle turn, but not so potent as DAPT which induced more OHCs in the apical turn

**Figure 4:**
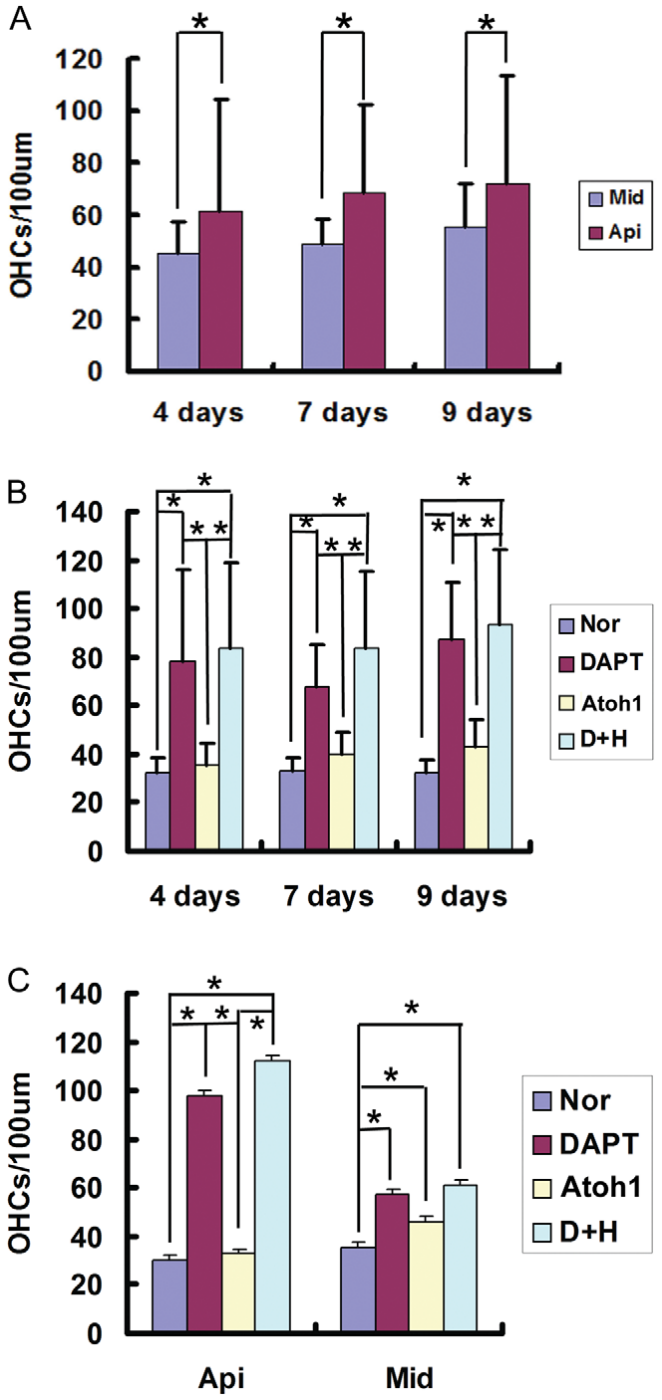
Statistical analysis of OHCs number after Atoh1 gene over expression and DAPT treatment. A. the number of OHCs increased with time, regardless of treatment (F = 10.961, P<0.001), furthermore, in the group of 7 and 9 days, the number of OHC in apical turn is higher than that of middle turn (the 95% confidence interval was listed in Table S2). The number of OHCs increased more significantly in the apical turn than that of the middle turn (F = 160.089, P<0.001). B: The effects of culture time and treatment on the number of OHCs. As is indicated in this chart, in the group of 4, 7, and 9 days, the numbers of OHCs treated by DAPT and DAPT+Atoh1 over expression are significantly than that of normal and solely Atoh1 over expression (the related 95% confidence interval was listed in Table S3). C: The effects of location and treatment on the number of OHCs. In the apical turn, the number of OHCs in DAPT and DAPT+Atoh1 over expression groups are much higher than that of normal and solely Atoh1 over expression group. In the middle turn, the number of OHCs in solely Atoh1 over expression was also significantly higher than that of normal groups (the related 95% confidence interval was listed in Table S4). These results indicated that DAPT could induce more extra OHCs in the apical turn than in middle turn. While in the middle turn, Atoh1 over expression could induce more extra OHCs than in apical turn. In this figure, Api: apical turn, Mid: Middle turn, Nor: Normal, DAPT: DAPT treat, Atoh1: Atoh1 over expression, D+A: DAPT treat plus Atoh1 over expression.

To investigate the effect of Atoh1 gene overexpression on the number of OHCs, we transfected cultured Organ of Corti from p0 rats with adv-Atoh1-EGFP for 24 hours. Atoh1 overexpressed in fibroblasts, spiral ganglion neurons, and the cells of the great epithelial ridge (GER) (Fig. S1 C, G, K, D, H, and L). Some cells in the sensory epithelia around the hair cells also expressed EGFP and Myosin VIIa simultaneously, indicating that Atoh1 was overexpressed in these cells (Fig. 1 C, D). After DAPT+ Atoh1 treatment, there were more than 4 rows of outer hair cells (sometimes even 5–7 rows) in the cultured Organ of Corti (Fig. 3D, D′). When we considered the single effects of DAPT treatment and Atoh1 overexpression, both induced an increase in the number of OHCs per 100 µm basilar membrane (DAPT treatment p<0.001, Atoh1 overexpression, p<0.001). Statistical analysis showed that the number of OHCs in the Atoh1 group increased significantly in the middle turn, but not in the apical turn (Fig. 4C). The number of hair cells in the group of DAPT treatment are far more than those with only Atoh1 overexpression (p<0.001). At 4, 7, and 9 days, the OHCs in the DAPT and DAPT+Atoh1 groups were significantly more than those in the control and Atoh1 overexpression alone groups (Fig. 4B). Simultaneously, DAPT induced more hair cells in the apical turn than in the middle turn (Fig. 4C).

### 3. The effects of inducing extra hair cell production of Atoh1 overexpression and DAPT treatment are time and location dependent. Their effects are additive but not synergistic

To assess the effects of culture time, hair cell location and the different treatments on the increase of OHC number, we performed a Games-Howell analysis using SPSS statistical software (version 18.0, SN: 5082047) (Tables S1, S2). The number of OHCs increased with time regardless of the treatment used (P = <0.001) (Fig. 4A, Table S1). Furthermore, in the samples observed after 7 and 9 days' culture, the number of OHCs was higher in the apical turn than in the middle turn (P<0.001) (Fig. 4A, Table S1). In the apical turn, the numbers of OHCs in the DAPT and DAPT+Atoh1 groups were much higher than those in the control (normal) and Atoh1 overexpression alone groups (Fig. 4C). These results indicate that their effects on the increase in the number of OHCs are time and location dependent.

**Figure 5:**
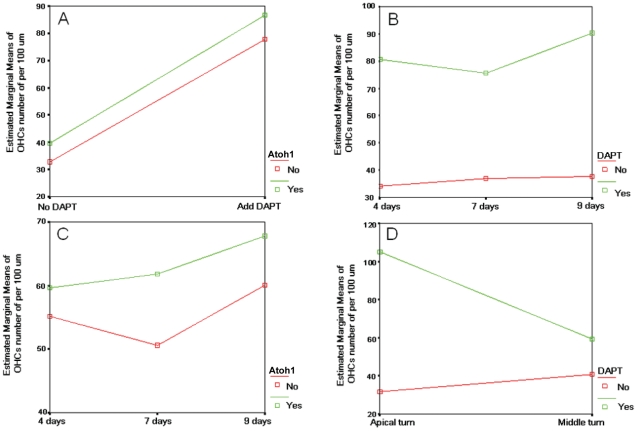
Interaction between treatment, culture time and hair cell location on the number of OHCs. A: The interaction curves are almost parallel, which means that their effects on the number increase of OHCs are independent. In other words, the number of OHCs increased similarly whether or not with Atoh1 over expression. B: In the group of 4, 7 and 9 days, the distance of the curves decreased first and then increased, which means that the single effect of on the number of OHCs is more significant during the 7^th^–9^th^ days of than that of 4^th^–7^th^ days. These result indicate that there were interaction between culture time and DAPT treatment. C: As for the case of Hath over expression, the distance between the two curves that represents with and without Atoh1 over expression increased during 4^th^ day–7^th^ day and then decreased during 7^th^–9^th^ day, which means that there was interaction between culture time and Hath over expression. First agonize before the 7^th^ day and then antagonize after the 7^th^ day. D: DAPT treatment could induce more extra OHCs in the apical turn than that of middle turn, which means that the location of the hair progenitors reacted differently to the treatment of DAPT. In A and C, No: no Atoh1 over expression. Yes: With Atoh1 over expression. In B andD: No: no DAPT treatment, Yes: with DAPT treatment.

The number of OHCs increased similarly after DAPT treatment no matter Atoh1 was overexpressed or not. As shown in Fig. 5A, the interaction curves of DAPT and Atoh1 are parallel. These results indicate that their effects on the increase in the number of OHCs are independent. When we considered the effects of DAPT and culture time, we noticed that the distance between the curves (with or without DAPT treatment) decreased at first and later increased from 4 to 7 and 9 days (Fig. 5B), which means that the effect of DAPT on the number of OHCs is more significant during days 7–9 than during days 4–7 (Fig. 5B). As for the effects of Atoh1 overexpression, the distance between the two curves representing the presence and absence of Atoh1 overexpression increased during days 4–7 and decreased during days 7–9, indicating that Atoh1 overexpression induced more hair cells during days 7–9 than during days 4–7 (Fig. 5C). When the interaction between DAPT treatment and location were considered, DAPT treatment induced more OHCs in the apical turn than in the middle turn, indicating that there may be more hair progenitors there that react to the DAPT treatment, or that the progenitors in the apical turn react better than those in the middle turn (Fig. 5D).

### 4. DAPT treatment changes the polarity of the stereocilia bundle of cultured hair cells

**Figure 6:**
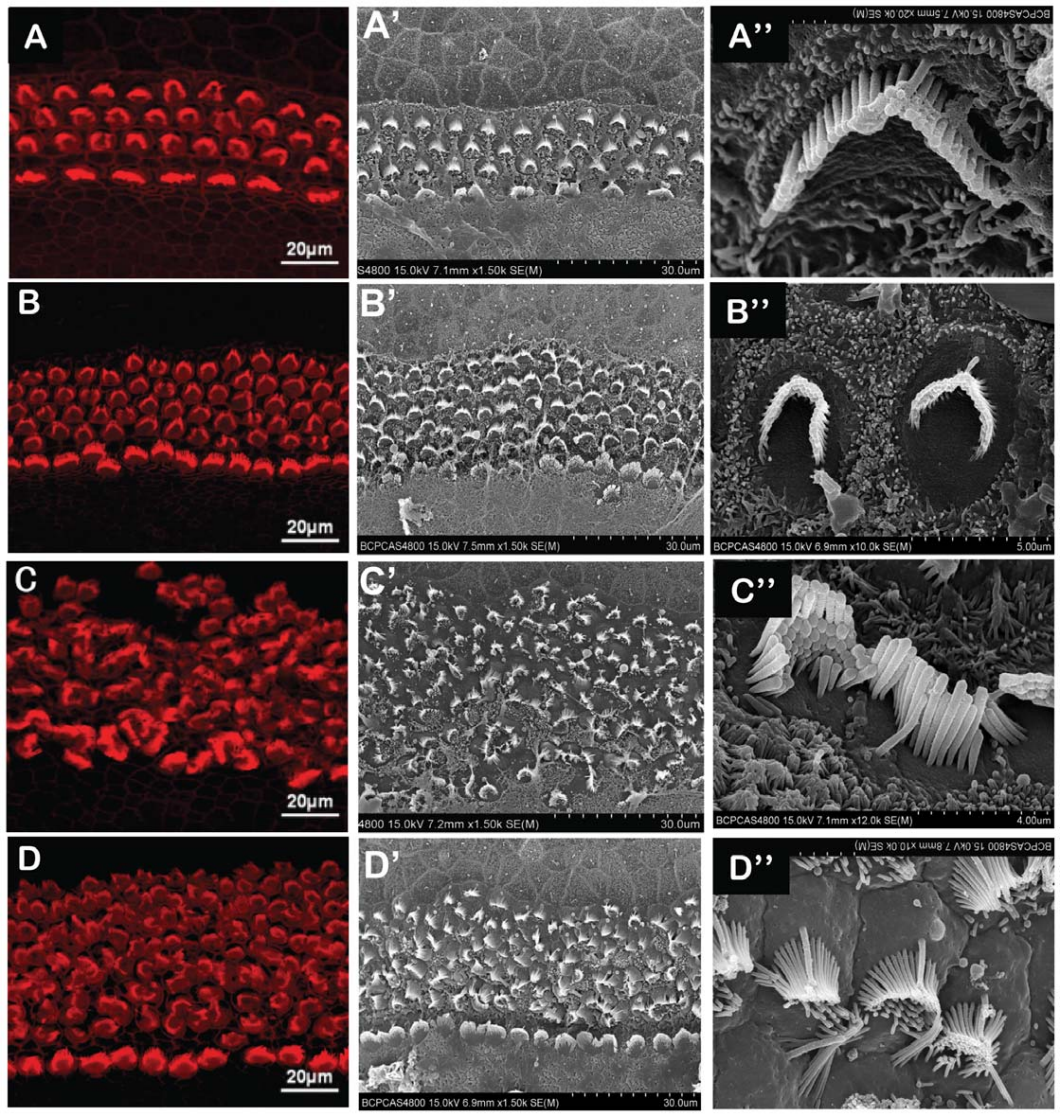
DAPT treatment changed the orientation of the stereocilia bundle of the cultured hair cells. A, B, C, D: the laser scanning confocal microscope image of the hair bundle on the hair cell (phalloidin staining); A′, B′, C′, D′: Scanning electron microscope image of the cultured hair cells; A and A′: without treatment, the stereocilia and hair cells bundle kept in order. B and B′: Atoh1 over expression group, the hair cell increased and their stereocilia bundles still kept relatively tidy. C and C′: Treated with DAPT, the orientation of the stereocilia bundle changed dramatically. The hair cells were out of order. D and D′: DAPT+ Atoh1 over expression, the stereociliary bundles were more untidy than control and solely Atoh1 over expression but tidier than that of treated solely with DAPT. A″, B″, C″, D″: Stereocilia bundle of an outer hair cell in the control group, Atoh1 over expression group, DAPT and DAPT+Atoh1 group separately.

When we observed the hair cells, we noticed that the orientation of stereociliary bundles changed in the DAPT, Atoh1 and DAPT+Atoh1 groups. To further investigate the ultra structural changes in the stereociliary bundles, we prepared samples from each group for scanning electron microscopy. The scanning electron microscope (SEM) and immunohistochemically staining using phalloidin under the laser scanning confocal microscope revealed that the stereociliary bundles underwent only modest changes in the control group (Fig. 6A, A′, A″). The stereociliary bundles in the DAPT group were totally disordered, crowded and no longer remained in rows (Fig. 2E, F; Fig. 6C, C′, C″). The shape of the stereociliary bundles on the OHCs varied, and the orientation of the stereocilia of the cultured IHCs and OHCs changed dramatically after DAPT treatment. Most of stereocilia bundles lost their ‘W’ shape and were irregularly shaped (Fig. 6C, C′). Some even turned 180° (Fig. 6 C″). The change in stereocilia orientation was noticeable on the fourth day after DAPT administration, and this change extended from the middle turn to the apical turn. When the cultured Organ of Corti samples were transfected with adv-Atoh1-EGFP, the hair cells were distributed more regularly than when treated with DAPT (Fig. 6B, B′, B″). In the group treated with DAPT and Atoh1 overexpression, the hair cells were distributed in a more orderly fashion than in the group treated solely with DAPT. However, the orientation of the stereociliary bundles was still clearly different than in the control and Atoh1 overexpression groups (Fig. 6D, D′, D″).

## Discussion

In this study, we investigated the effects of Atoh1 overexpression and Notch signaling pathway inhibition using treatment with the r-secretase inhibitor DAPT on the generation of extra hair cells in cultured Organ of Corti isolated from newborn rats. The results showed that both DAPT treatment and Atoh1 overexpression were able to induce the generation of extra hair cells on the basilar membrane. However, the induction of extra hair cells by these two methods to is additive rather than synergistic. Furthermore, we noticed for the first time that DAPT treatment caused the orientation of the stereocilia bundle on hair cells to change dramatically, and the overexpression of Atoh1 was antagonistic to DAPT treatment in this regard.

### 1. The extra hair cells that appeared after DAPT treatment may be derived from immature supporting cells in the Organ of Corti of newborn animals

The Notch signaling pathway plays important roles in the development of the inner ear and the differentiation of hair cells and supporting cells through lateral inhibition [21], [22], [23]. When the Notch receptor binds to its ligands, r-secretase involves in the activation of Notch signaling by producing the Notch intracellular domain (NICD). The NICD then enters the nucleus and binds with the DNA-binding protein CSL and the coactivator protein Mastermind. This initiates the expression of downstream genes such as Hesl, Hes5, Hesrl, and BLBP (brain lipid-binding protein). Hes1 and Hes5 are inhibitory and bind to Atoh1, a bHLH transcription factor that plays a key role in inducing progenitor cells to differentiate into hair cells [1], [14], [24], [25], [26], [27], [28].

DAPT, as an r-secretase inhibitor, blocks the Notch signaling pathway and reduces the expression levels of Hes1 and Hes5, thereby removed their suppression on Atoh1 expression. As a consequence, Atoh1 promoted the differentiation of hair cells. Several studies have revealed that inhibiting the Notch signaling pathway with different inhibitor in immature cochlear sensory epithelial cells can increase the number of hair cells. Beside DAPT, which has been used by Takebayashi et al in increasing hair cells that derived from immature supporting cells [18], MDL-28170 was also used to inhibit the Notch signaling pathway by Yamamoto group and Hori [29] group in inducing extra hair cells [19]. Kiernan et al (2005) used gene knock out animal model and demonstrated that in the Dll1hyp/− Jag2−/− mice, hair cells arised 1.6 folds [27]. Alternatively, antisense oligonucleotides (AS-Notch) was used by Zien et al to down regulate the Notch signal in cultured Organ of Corti at E16, P0, and P3 for 5 days and found that both IHCs and OHCs were increased in the E16 group in the middle turn of the Organ of Corti [30]. Our results are consistent with theirs in that when Notch signaling pathway was attenuated the hair cells increased and that the extra hair cells came from supporting cells. Our results further showed that when we treated cultured Organ of Corti from P0 rats, extra hair cells emerged in both the middle and apical turns. Moreover, the increase in the number of hair cells was more obvious in the apical turn than in the middle turn. Given that the Organ of Corti matures from the basal turn towards the apical turn in the newborn animal and that the increase in the number of hair cells was more obvious in the apical turn than in the middle turn, these results suggest that the apical turn is more sensitive to DAPT treatment and that the reaction depends on the maturity of the organ. As some studies have shown that Notch1 and Hes5 express on outer supporting cells during the late embryonic and newborn stages [31], we postulate that in our experiments DAPT treatment increased the number of hair cells by inhibiting the Notch signaling pathway and its lateral inhibition effect on supporting cell differentiation, which then resulted in the conversion of some immature supporting cells into hair cells. We also provided the direct evidence of trans-differentiation of supporting cells to hair cells (Fig. 3 F).

### 2. Atoh1 overexpression and DAPT may works on different group progenitor cells in inducing extra hair cells in cultured postnatal Organ of Corti

Zheng and Gao [16] overexpressed Atoh1 in the Organ of Corti by electroporation and obtained extra hair cells in the GER region, which suggested that there might be some progenitor cells in that area. In our experiments, we noticed that the extra hair cells induced by Atoh1 overexpression appeared not only in the GER region but also in the OHC region in cultured postnatal Organ of Corti. In other words, some extra hair cells may come from the progenitors that reside in the region of Organ of Corti and GER. These differences might be attributed to the different methods use by them and us. Zheng [16] overexpressed Math1 gene by electroporation, while in our case, Atoh1 gene was delivered by adenovirus to the cultured Organ of Corti, which may result in the expression in different progenitor cells. We currently don't know what cells are these progenitors. Further study may be helpful in defining the progenitors that resides in the region of the Organ of Corti.

One thing interesting is that in the Atoh1 overexpression group, there were more extra hair cells in in the middle turn than in the apical turn, while more extra hair cells were induced by DATP treatment in the apical turn than that of middle turn. These results suggest that the progenitors that reacts to Atoh1 overexpression mainly lies in the middle turn and that those progenitors that reacts to DAPT treatment mainly lies in the apical turn. As discussed above, the extra hair cells in the group mainly came from supporting cells neighboring to original hair cells which exert lateral inhibition through Notch pathway, while the progenitors that could differentiation into hair cells may not be limited to supporting cells. Therefore, We postulate that those cells that overexpressed the Atoh1 gene may not be the same population as those affected by DAPT treatment, which may contribute to the additive but not synergistic effect in extra hair cells induction from Atoh1 overexpression and DAPT treatment.

### 3. Disturbance of Notch signaling pathway may affects the arrangement of hair cells and their hair bundles

Although both Atoh1 overexpression and DAPT treatment were able to induce extra hair cells in cultured Organ of Corti from newborn animals, their effects on the orientations of the hair cells stereocilia bundles were different. In our results, we noticed that DAPT treatment resulted in the loss of the polarity of the stereocilia on most of the hair cells. The arrangement of the stereocilia was drastically changed that their orientation lost its normal pattern, which means that the opening direction of stereocilia bundles on almost all of the hair cells were affected. Our results are in accordance with those of Kiernan et al [27] in that the polarity of the hair bundles was also changed drastically in the Dll1hyp/− Jag2−/− mice cochleae. Doetzlhofer et al [32] have also treated neonatal Organ of Corti with DAPT and showed that the arrangement of hair cells has considerably changed. All these results demonstrated that the disturbance of Notch pathway in the developing Organ of Corti would affect the arrangement of hair cells as well as the polarity of their hair bundles. As it is well known that planar cell polarity (PCP) pathway determined the arrangement of cells and the orientation of hair bundles in mammalian auditory sensory organ [33], we postulate that the normal function of Notch pathway may be necessary for the action of PCP pathway in the development of inner ear sensory epithelia. When the Notch signal was blocked by r-secretase inhibitor, the new hair cells trans-differentiated from supporting cells might contributes to the disarrangement of hair bundles because they also brought up new hair bundles but not in the same row with the original hair cells. When we compared the hair bundle shape in the Atoh1 overexpression groups with those treated by DAPT, we noticed that the orientation of the stereocilia did not change so drastic and were kept relatively more regular, which may be attributed to the less increase of new hair cells inducted by Atoh1 overexpression. We don't know the exact mechanism behind these changes. Further studies on related genes' expression may reveal how these two factors work in arranging the hair cells and their stereociliary bundles.

## Materials and Methods

Neonatal Sprague-Dawley (SD) rats (P0) of both genders were purchased from the Beijing HFK Bio-Technology Co. Ltd Affiliated to the Institute of Laboratory Animal Sciences at the Chinese Academy of Medical Sciences (CAMS). The Organ of Corti of each animal (both sides) were dissected, cultured in Petri dishes and treated with the r-secretase inhibitor DAPT or transfected with adenovirus- Atoh1-EGFP vector in each group. The groups were determined as follow: 1) In the control group, no treatment was given to the cultured samples. 2) In the DAPT group, 5 µM (final concentration) DAPT (Sigma) was added to the culture medium daily from the start of the experiment until the specimens were picked for laser scan confocal microscopy and electron microscopic observation. 3) In the Atoh1 group, the cultured Organ of Corti were transfected with the adenovirus- Atoh1-EGFP vector (abbreviated as adv-Atoh1-EGFP) for the first 24 h, and the medium was changed to the same medium as that used for the control group thereafter. Adv-Atoh1-EGFP was constructed in the same manner as that of Zheng and Gao [16] to overexpress the Atoh1 gene. 4) In the DAPT+Atoh1 group, both DAPT treatment and adenovirus- Atoh1-EGFP vector transfection were used. The durations of the DAPT/adv-Atoh1-EGFP treatments were the same as those used in the DAPT group/Atoh1 group respectively. The cultured specimens were then fixed, and Myosin VIIa immunohistochemistry was carried out before observation under laser scanning confocal microscope. To count the number of hair cells, we stained the stereociliary bundles on the top of each hair cell with phalloidin and observed the cells under a laser scanning confocal microscope. The number of IHCs and OHCs were counted at 4, 7 and 9 days. Five Organs of Corti were evaluated at each time point. The care and use of the animals and the experimentation were monitored and approved by the the Institutional Animal Care, Use & Ethics Committee of PLA General Hospital. The permit numbers for this study is No. 20080156.

### 1. Culturing of Organ of Corti from neonatal SD rats in vivo

P0 SD rats were anesthetized by freezing at −20°C for 5 min and then sterilized by immersion in ethanol. The animals were then decapitated, and their crania were cut along the middle line. The outer skin, soft tissue, brain and other contents were removed quickly, and the bilateral temporal bones were isolated and immediately immersed in Leibovitz's L-15 Medium (Gibco). The cochlear outer wall was opened and removed under a dissecting microscope (Olympus). The entire basilar membrane was exposed, isolated from the modiolus and transferred to another Petri dish containing L15 Leibovitz's medium. The spiral ligament and stria vascularis were peeled off with gossamer tweezers. The whole procedure was finished within 10 minutes.

### 2. Tissue culture and treatment

The isolated basilar membrane samples were cultured in six-well plates. A sterilized cover slip was put into each well in advance. Two drops of DMEM (containing 4.5 g/L D-glucose, Gibco) were added and the isolated basilar membrane was put into the DMEM medium to adhere to the bottom with the tectorial membrane and Reissner's membrane facing up. Intensive attentions were paid during this procedure, as we noticed that the extra rows of hair cell may come from the basilar membrane if not extended well [20]. Ten minutes later, 1 ml of DMEM containing 10% FBS (Gibco, U.S.A.) was carefully and slowly added to the medium, and the culture plate was put into a 37°C, 5% CO2 incubator. The medium was replaced every day with DMEM containing 5% FBS. The treatment of all groups was carried out as described above. For the DAPT and DAPT+Atoh1 groups, DAPT was freshly prepared and added each day when the culture medium was changed. For the Atoh1 and Dapt+Atoh1 groups, the final titer of adv-Atoh1-EGFP was 1∶400 and the cells in the Organ of Corti were transfected for 24 hours. The gamma-secretase inhibitor DAPT was obtained from Tocris Bioscience.

### 3. Immunofluorescence

The basilar membrane specimens were rinsed with 0.1 M PBS three times and fixed with 4% paraformaldehyde at 4°C for 30 min. The samples were then rinsed three times in 0.1 M PBS for 5 min each time. The specimens were then soaked in 0.1% PBST for 30 min. After blocking the antigen with 5% immune serum for 30 min and rinsing three times in PBS, a Myosin VIIa primary antibody (rabbit anti-rat, Santa Cruz Biotechnology, U.S.A., diluted 1∶200 in PBS) was added to the basilar membrane to label the hair cells. After washing three times with PBS, fluorescein-labeled secondary antibody (Invitrogen, U.S.A., Goat anti-rabbit, A11008) was added and then incubated in the dark at room temperature for 1 hour. After washing with 0.1% PBST three times, phalloidin (Sigma, U.S.A.) was added and the specimens were kept in the dark for 30 min. Finally, the sections were mounted using anti-Fade Dapi-fluromount-G (SouthernBiotech, USA) and observed under a confocal microscope (Zeiss AXIOVERT 200 MBP, Germany).

### 4. Transmission and scanning electronic microscopy

#### Semi-thin sectioning and transmission electron microscopy

Cultured Organ of Corti specimens from each of the groups were fixed with 2.5% glutaraldehyde and maintained at 4°C for 24–48 hours. The specimens were then washed in phosphate buffer and post-fixed in 1% osmium tetroxide. After dehydration in a graded series of ethanol dilutions and embedding in Epon 812 resin, serial sections (2 µm thick, semi-thin) were made perpendicular to the basilar membrane, stained with toluidine blue and examined under a light microscope. For evaluation by transmission electron microscopy, the cochlear epithelia were dehydrated in an ascending ethanol series and embedded in Epon 812 resin. Sections were taken using an LKB-V ultramicrotome. Ultrathin sections (approximately 70 nm thick) were taken serially, placed in order on 230-mesh copper grids and stained with uranyl acetate and lead citrate. The specimens were observed using an HITACHI H-7650 transmission electron microscope.

#### Scanning electron microscopy

To observe the hair cell stereocilia, cultured Organ of Corti from each group were prepared for scanning electron microscopic observation. Briefly, the specimens were washed three times in phosphate buffer, fixed with 2.5% glutaraldehyde, stored at 4°C for 24–48 hours and postfixed in 1% osmium tetroxide. After dehydration in a graded series of ethanol dilutions ethanol, the specimens were critical-point dried (CO_2_) using an HCP-2 critical point dryer and mounted on aluminum stubs with silver paint. Gold/palladium was sputter-coated on the specimens using an E-102 ion sputter for viewing under an HITACH S-4800 Scanning electron microscope (15 kV accelerating voltage).

### 5. Cell counting and statistical analysis

The numbers of IHCs and OHCs in the cultured Organ of Corti (per 100-micrometer length along the basilar membrane, regardless of the width) were counted. They were stained with phalloidin to distinguish the stereocilia bundle on each hair cell. Statistical analysis was carried out using SPSS software (version 18.0, SN: 5082047). The Factorial design ANOVA (two-tailed) analytical method was used for analysis. Games-Howell analysis was performed to assess the effects of culture time, hair cell location and the different treatments on the increase in OHC number.

## Supporting Information

Figure S1 **Culture and treatment of the P0 rat cochlear basilar membrane.** A, E, I: Image of cultured Organ of Corti without any treatment at 4, 7 and 9 days; B, F, J: the cultured Organ of Corti treated with DATP for 4, 7 and 9 days; C, G, K: the cultured Organ of Corti with Hath1 over expression for 4, 7 and 9 days; D, H, L: the cultured Organ of Corti with Hath1 over expression plus DAPT treatment for 4, 7 and 9 days; red fluorescence: Myosin VIIa, green fluorescence: EGFP.
(TIF)

Table S1 **Tests of between-subjects effects on inducing extra outer hair cells and Interaction between each groups.**
(DOC)

Table S2 **The effects of culture time and location of OHCs on the number of OHCs.**
(DOC)

Table S3 **The effects of culture time and treatment on the number of OHCs.**
(DOC)

Table S4 (DOC)
