# Supplementary figures and images for: Effects of DAPT and Atoh1 Overexpression on Hair Cell Production and Hair Bundle Orientation in Cultured Organ of Corti from Neonatal Rats
Source: PLoS One. 2011 Oct 20;6(10):e23729. doi: 10.1371/journal.pone.0023729 (PMC3197578; doi:10.1371/journal.pone.0023729)

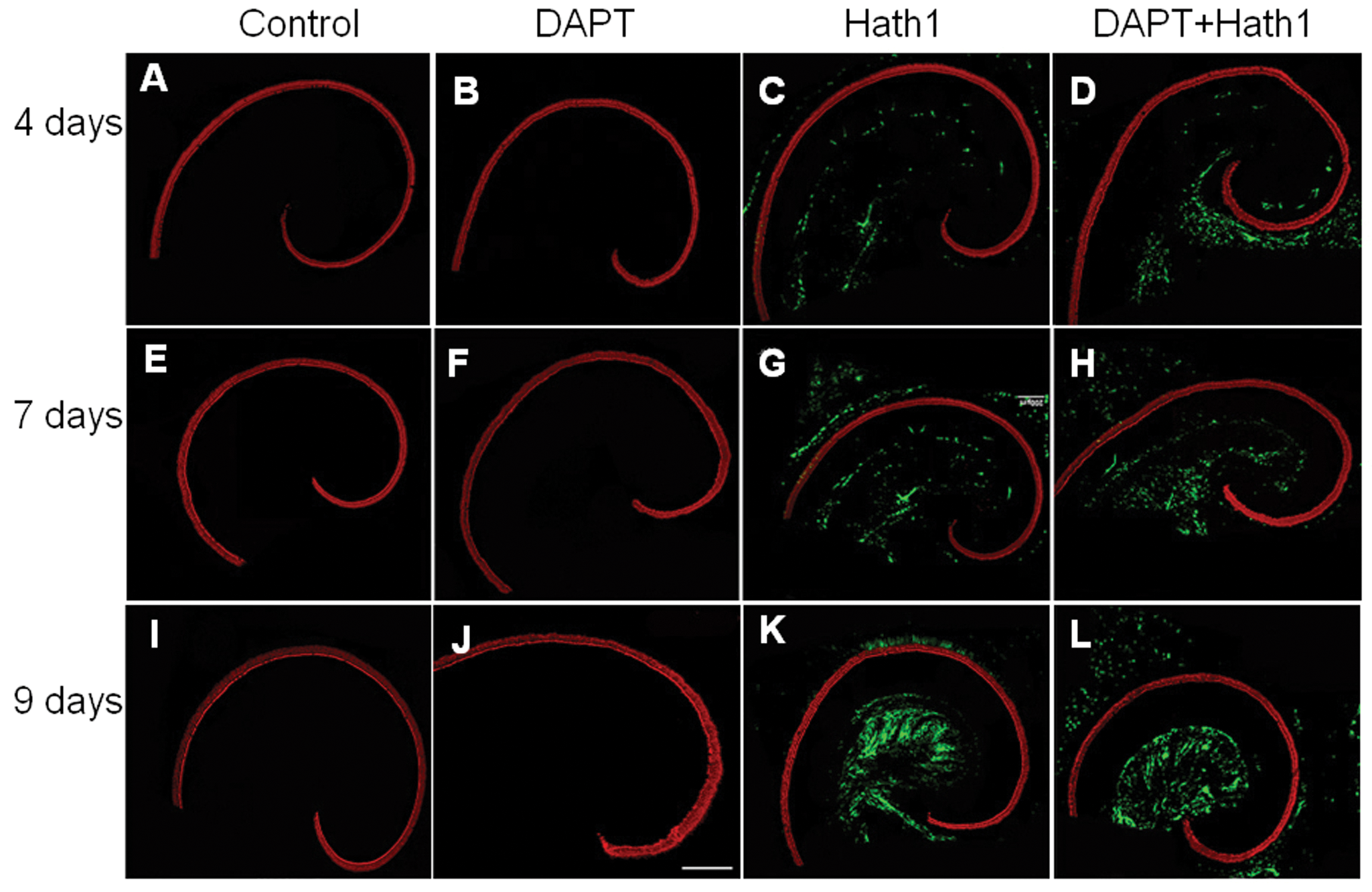

Supplement: Figure S1 — Culture and treatment of the P0 rat cochlear basilar membrane. A, E, I: Image of cultured Organ of Corti without any treatment at 4, 7 and 9 days; B, F, J: the cultured Organ of Corti treated with DATP for 4, 7 and 9 days; C, G, K: the cultured Organ of Corti with Hath1 over expression for 4, 7 and 9 days; D, H, L: the cultured Organ of Corti with Hath1 over expression plus DAPT treatment for 4, 7 and 9 days; red fluorescence: Myosin VIIa, green fluorescence: EGFP. (TIF) [file pone.0023729.s001.tif]
