# Supplementary material for: Effects of DAPT and Atoh1 Overexpression on Hair Cell Production and Hair Bundle Orientation in Cultured Organ of Corti from Neonatal Rats
Source: PLoS One. 2011 Oct 20;6(10):e23729. doi: 10.1371/journal.pone.0023729 (PMC3197578; doi:10.1371/journal.pone.0023729)
Supplement: Table S1 — Tests of between-subjects effects on inducing extra outer hair cells and Interaction between each groups. (DOC) [file pone.0023729.s002.doc]

## Supporting Information:

## Table S1. Tests of Between-Subjects Effects on inducing extra outer hair cells and Interaction between each groups

| Source | Type III Sum of Squares | df | Mean Square | F | Sig. | Partial Eta Squared | Noncent. Parameter | Observed Power(a) |
| --- | --- | --- | --- | --- | --- | --- | --- | --- |
| Corrected Model | 303730.650(b) | 12 | 25310.887 | 124.261 | .000 | .811 | 1491.133 | 1.000 |
| Intercept | 1260124.706 | 1 | 1260124.706 | 6186.449 | .000 | .947 | 6186.449 | 1.000 |
| Culture time | 4183.955 | 2 | 2091.978 | 10.270 | .000 | .056 | 20.541 | .987 |
| location | 30555.015 | 1 | 30555.015 | 150.007 | .000 | .302 | 150.007 | 1.000 |
| DAPT | 190625.927 | 1 | 190625.927 | 935.858 | .000 | .730 | 935.858 | 1.000 |
| HATH1 | 5509.216 | 1 | 5509.216 | 27.047 | .000 | .072 | 27.047 | .999 |
| DAPT * HATH1 | 121.009 | 1 | 121.009 | .594 | .441 | .002 | .594 | .120 |
| Culture time * DAPT | 2894.959 | 2 | 1447.480 | 7.106 | .001 | .039 | 14.212 | .930 |
| Culture time * HATH1 | 684.878 | 2 | 342.439 | 1.681 | .188 | .010 | 3.362 | .353 |
| location * DAPT | 69128.213 | 1 | 69128.213 | 339.378 | .000 | .494 | 339.378 | 1.000 |
| location* HATH1 | 27.477 | 1 | 27.477 | .135 | .714 | .000 | .135 | .066 |
| Error | 70680.823 | 347 | 203.691 |  |  |  |  |  |
| Total | 1634536.178 | 360 |  |  |  |  |  |  |
| Corrected Total | 374411.472 | 359 |  |  |  |  |  |  |

* Dependent Variable: the number of OHCs per 100um along the basilar membrane

a Computed using alpha = .05

b R Squared = .811 (Adjusted R Squared = .805).

From this char we can see that according to the statistical significant standard, p<0.05, the main effect of DPAT treatment and Hath1 over expression on the number of OHCs were different. There were interactions between DAPT and cultured days, location of OHCs and DAPT, but no interaction between DAPT and Hath1 over expression, as well as between days and Hath1 over expression.
