# Supplementary material for: Effects of DAPT and Atoh1 Overexpression on Hair Cell Production and Hair Bundle Orientation in Cultured Organ of Corti from Neonatal Rats
Source: PLoS One. 2011 Oct 20;6(10):e23729. doi: 10.1371/journal.pone.0023729 (PMC3197578; doi:10.1371/journal.pone.0023729)
Supplement: Table S2 — The effects of culture time and location of OHCs on the number of OHCs. (DOC) [file pone.0023729.s003.doc]

## Table S2: The effects of culture time and location of OHCs on the number of OHCs.

| Cultured time | Location of OHCs | Mean | Std. Error | 95% Confidence Interval | |
| --- | --- | --- | --- | --- | --- |
|  |  |  |  | Lower Bound | Upper Bound |
| 4 day | apical turn | 69.348 | 1.784 | 65.840 | 72.857 |
|  | middle turn | 45.437 | 1.784 | 41.929 | 48.946 |
| 7 day | apical turn | 63.437 | 1.784 | 59.929 | 66.945 |
|  | middle turn | 48.895 | 1.784 | 45.386 | 52.403 |
| 9 day | apical turn | 72.344 | 1.784 | 68.836 | 75.852 |
|  | middle turn | 55.521 | 1.784 | 52.013 | 59.029 |

Dependent Variable: number of OHCs per 100 um Organ of Corti on the basilar membrane
