# Supplementary material for: Effects of DAPT and Atoh1 Overexpression on Hair Cell Production and Hair Bundle Orientation in Cultured Organ of Corti from Neonatal Rats
Source: PLoS One. 2011 Oct 20;6(10):e23729. doi: 10.1371/journal.pone.0023729 (PMC3197578; doi:10.1371/journal.pone.0023729)
Supplement: Table S3 — The effects of culture time and treatment on the number of OHCs. (DOC) [file pone.0023729.s004.doc]

## Table S3 The effects of culture time and treatment on the number of OHCs.

| Cultured time | Treatment | Mean | Std. Error | 95% Confidence Interval | |
| --- | --- | --- | --- | --- | --- |
|  |  |  |  | Lower Bound | Upper Bound |
| 4 day | normal | 32.403 | 2.522 | 27.441 | 37.364 |
|  | DAPT group | 77.915 | 2.522 | 72.954 | 82.877 |
|  | Hath1 group | 35.715 | 2.522 | 30.753 | 40.676 |
|  | DM group | 83.538 | 2.522 | 78.577 | 88.500 |
| 7 day | normal | 33.415 | 2.522 | 28.454 | 38.377 |
|  | DAPT group | 67.692 | 2.522 | 62.731 | 72.654 |
|  | Hath1 group | 40.140 | 2.522 | 35.179 | 45.102 |
|  | DM group | 83.415 | 2.522 | 78.454 | 88.377 |
| 9 day | normal | 32.643 | 2.522 | 27.681 | 37.604 |
|  | DAPT group | 87.442 | 2.522 | 82.480 | 92.404 |
|  | Hath1 group | 42.599 | 2.522 | 37.638 | 47.561 |
|  | DM group | 93.047 | 2.522 | 88.085 | 98.008 |

Dependent Variable: number of OHCs per 100 um Organ of Corti on the basilar membrane
