# Supplementary material for: Effects of DAPT and Atoh1 Overexpression on Hair Cell Production and Hair Bundle Orientation in Cultured Organ of Corti from Neonatal Rats
Source: PLoS One. 2011 Oct 20;6(10):e23729. doi: 10.1371/journal.pone.0023729 (PMC3197578; doi:10.1371/journal.pone.0023729)
Supplement: Table S4 — (DOC) [file pone.0023729.s005.doc]

## Table S4

| treatment | location | Mean | Std. Error | 95% Confidence Interval | |
| --- | --- | --- | --- | --- | --- |
|  |  |  |  | Lower Bound | Upper Bound |
| normal | apical turn | 30.223 | 2.059 | 26.172 | 34.274 |
|  | middle turn | 35.417 | 2.059 | 31.366 | 39.469 |
| DAPT group | apical turn | 98.153 | 2.059 | 94.102 | 102.204 |
|  | middle turn | 57.213 | 2.059 | 53.162 | 61.264 |
| Hath1 group | apical turn | 32.793 | 2.059 | 28.742 | 36.844 |
|  | middle turn | 46.176 | 2.059 | 42.125 | 50.228 |
| DM group | apical turn | 112.337 | 2.059 | 108.285 | 116.388 |
|  | middle turn | 60.997 | 2.059 | 56.946 | 65.048 |

**Dependent Variable: number of OHCs per 100 um Organ of Corti on the basilar membrane**
